# Acute retroviral syndrome and high baseline viral load are predictors of rapid HIV progression among untreated Argentinean seroconverters

**DOI:** 10.1186/1758-2652-14-40

**Published:** 2011-08-10

**Authors:** M Eugenia Socías, Omar Sued, Natalia Laufer, María E Lázaro, Horacio Mingrone, Daniel Pryluka, Carlos Remondegui, María I Figueroa, Carina Cesar, Ana Gun, Gabriela Turk, María B Bouzas, Ravi Kavasery, Alejandro Krolewiecki, Héctor Pérez, Horacio Salomón, Pedro Cahn

**Affiliations:** 1 Hospital J.A. Fernández, Cerviño 3356, Buenos Aires, Argentina; 2 Fundación Huésped, Peluffo 3932, Buenos Aires, Argentina; 3 Nexo Asociación Civil, Callao 339, Buenos Aires, Argentina; 4 Hospital Zonal Ramón Carrillo, Moreno 601, Bariloche, Argentina; 5 Hospital Muñiz, Uspallata 2272, Buenos Aires, Argentina; 6 MEDICUS, Azcuénaga 870, Buenos Aires, Argentina; 7 Hospital San Roque, San Martín 330, San Salvador de Jujuy, Argentina; 8 Centro Nacional de Referencia para el SIDA, Universidad de Buenos Aires, Paraguay 2155, Buenos Aires, Argentina; 9 Yale University School of Medicine, 333 Cedar Street, New Haven, Connecticut, USA

## Abstract

**Background:**

Diagnosis of primary HIV infection (PHI) has important clinical and public health implications. HAART initiation at this stage remains controversial.

**Methods:**

Our objective was to identify predictors of disease progression among Argentinean seroconverters during the first year of infection, within a multicentre registry of PHI-patients diagnosed between 1997 and 2008. Cox regression was used to analyze predictors of progression (LT-CD4 < 350 cells/mm^3^, B, C events or death) at 12 months among untreated patients.

**Results:**

Among 134 subjects, 74% presented with acute retroviral syndrome (ARS). Seven opportunistic infections (one death), nine B events, and 10 non-AIDS defining serious events were observed. Among the 92 untreated patients, 24 (26%) progressed at 12 months versus three (7%) in the treated group (p = 0.01). The 12-month progression rate among untreated patients with ARS was 34% (95% CI 22.5-46.3) versus 13% (95% CI 1.1-24.7) in asymptomatic patients (p = 0.04). In univariate analysis, ARS, baseline LT-CD4 < 350 cells/mm^3^, and baseline and six-month viral load (VL) > 100,000 copies/mL were associated with progression. In multivariate analysis, only ARS and baseline VL > 100,000 copies/mL remained independently associated; HR: 8.44 (95% CI 0.97-73.42) and 9.44 (95% CI 1.38-64.68), respectively.

**Conclusions:**

In Argentina, PHI is associated with significant morbidity. HAART should be considered in PHI patients with ARS and high baseline VL to prevent disease progression.

## Background

Cohort studies addressing primary HIV infection (PHI) have been used as a tool to study the natural history of HIV and to estimate the incidence of AIDS-defining events, as well as other non-associated AIDS comorbidities. It is increasingly recognized that early host-virus interactions may influence the later course of disease [1,2]. Therefore, follow up of patients immediately after seroconversion may help identify prognostic markers useful in the evaluation of therapeutic approaches.

To date, most studies of HIV seroconverters have been performed in Europe or North America [3-5]. Scarce information exists on this issue from resource-limited settings, particularly in South America, where there are different host, social and viral (i.e., subtype) characteristics that may alter the course of HIV infection [6-8].

In Argentina, it is estimated that there are approximately 130,000 persons living with HIV/AIDS, but only half of them are aware of their status. In 2008, more than 4000 new HIV infections were reported [9]. However, information regarding patients diagnosed during the early stages of infection is limited. To address this situation, a multicentre registry of patients with primary HIV infection in Argentina was started in 2008 [10,11].

This paper describes the epidemiological, clinical, immunological and virological characteristics of the first 134 patients enrolled in our cohort with the aim of identifying potential markers associated with HIV progression.

## Methods

### Study population

*Grupo Argentino de Seroconversión *[10,11] is an ongoing multicentre Argentine observational cohort of patients diagnosed during primary HIV infection. This cohort was started in 2008 and includes two data sets: the first one includes patients diagnosed between 1997 and 2007, and the second prospectively follows patients diagnosed after January 2008.

Inclusion criteria for enrolment in the cohort are: age > 16 years at first evaluation, confirmed diagnosis of primary HIV infection, and first medical and laboratory evaluation (i.e., CD4 cell count and plasma HIV RNA) within six months of the probable date of infection. Primary HIV infection is defined as: (1) detection of HIV RNA or p24 antigen with a simultaneous negative or indeterminate Western blot assay [12]; or (2) positive Western blot with a negative test within the previous six months Hence, it includes both acute and recent HIV-infection patients.

Structured questionnaires are used for baseline and follow-up visits. Clinical and laboratory information is updated every six months until death or loss to follow up.

In this paper, we report on patients who were diagnosed up to 31 December 2008. Analysis of disease progression was limited to the first year of infection.

### Ethical considerations

The *Grupo Argentino de Seroconversión *study protocol was approved by the Huésped Foundation Ethics Committee. All patients followed prospectively signed written informed consent before enrolment. Patients studied retrospectively signed consent at their first follow-up visit, if still alive.

### Definitions

We defined PHI as "symptomatic" if one or more symptoms associated with acute retroviral syndrome were present [13,14]. "Severe symptomatic PHI" was defined as presence of B or C events, (according to the Centers for Disease Control and Prevention 1993 classification [15]), any other serious non-AIDS-related events, or death at the time of HIV seroconversion.

In symptomatic patients, the date of infection was estimated as 14 days before the onset of symptoms. In asymptomatic patients, the date of infection was estimated as the midpoint between the last negative and the first positive test or one month before the date of the indeterminate or negative Western blot assay [16-18].

HIV progression was defined either by clinical (B or C events [15]), or immunological (CD4 cell count < 350 cells/mm^3^) criteria, whichever occurred first. We chose these endpoints based on the current national and international recommendations for initiation of antiretroviral therapy [19,20]. Analysis of disease progression was limited to those patients who did not start treatment within the first 120 days of infection.

### Statistical analysis

Quantitative variables were described using mean and standard deviation (SD) in cases where the underlying distribution was normal; median and interquartile ranges (IQR) were used for variables without normal distribution. Differences were analyzed using Student's t-test for independent samples or the non-parametric Wilcoxon Rank Sum test.

Categorical variables were described using proportions and percentages. Differences between proportions were analyzed with the Chi-square test, or Fisher's exact test. Differences were considered statistically significant for p < 0.05, two-tailed tests. Univariate analysis was performed for the variables hypothesized as risk factors for events under study. All the variables of interest for the study were included in the multivariate analysis. Cox regression analysis was performed and the hazard risk (HR), 95% confidence interval (CI) and p value were calculated for each variable.

Progression-free survival time was measured from the estimated date of infection to the date of progression. For those patients who did not experience an event, data was censored at their last visit within their first year of infection or at treatment initiation. Time until an event was studied using Kaplan-Meier survival analysis, and the log rank test was applied for significance. Overall median time estimates, as well as median time by arm and corresponding 95% CI, are given. Kaplan-Meier plots are shown. Data analysis was performed with SPSS 15.0, 2007 (Chicago, Illinois).

## Results

### Baseline characteristics

As of December 2008, 134 patients with primary HIV infection were enrolled in the cohort; 99 retrospectively and 35 prospectively. Baseline characteristics are summarized in Table 1. Most patients were male (n = 109) with a median age of 32 years (IQR 25-39). More than half of the patients (53%) defined themselves as men who have sex with men (MSM), while 50 (37%) reported heterosexual exposure. Only one patient reported intravenous drug use as the probable route of infection.

**Table 1:** Baseline characteristics of *Grupo Argentino de Seroconversión *cohort (N = 134)

Characteristic	All (N = 134)	Symptomatic PHI		p
			
		YES (n = 99)	NO (n = 35)	
Age at HIV diagnosis, mean years (SD)	33.4 (10.7)	33.8 (10.37)	32.2 (11.64)	0.44

Male sex, n (%)	109 (81.3)	79 (79.8)	30 (85.8)	0.61

High school education or more, n (%)	79 (75.2)	59 (72.8)	20 (83.4)	0.3

Born in Buenos Aires, n (%)	74 (67.9)	56 (67.5)	18 (69.2)	0.61

Employed, n (%)	82 (70.7)	62 (70.5)	20 (71.4)	0.89

Reason for HIV test, n (%)				

Physician's suspicion	61 (48.4)	56 (59.6)	5 (15.6)	**< 0.001**

Patient request	42 (33.3)	27 (28.7)	15 (46.9)	

Routine	23 (18.3)	11 (11.7)	12 (37.5)	

Risk factor for HIV transmission, n (%)				

MSM	71 (53)	51 (51.5)	20 (57.1)	0.788

Heterosexual	50 (37.3)	38 (38.4)	12 (34.3)	

IDU	1 (0.7)	1 (1)	0 (0)	

Missing	12 (9)	9 (9)	3 (8.6)	

HIV RNA, median log_10 _copies/mL (IQR)	4.87 (4.11-5.51)	5.12 (4.49-5.69)	4.36 (3.43-4.95)	**< 0.001**

CD4 cell count, median cells/mm^3^ (IQR)	479 (341-682)	466 (327-609)	533 (425-814)	**0.019**

HAART initiation, n (%)	42 (31.3)	39 (39.4)	3 (8.6)	**0.003**

MSM-men who have sex with men; IDU-injection drug user; HAART-highly active antiretroviral therapy

Most of the patients (n = 74) were from Buenos Aires city and its surroundings suburbs, areas that concentrate 44% of people living with HIV/AIDS in Argentina [9]. Seventy-five percent of patients completed at least high school and 29% were unemployed. HIV testing was requested based on a physician's clinical suspicion in 48% of cases and because of patient's request in 33% of cases. In 18% of cases, HIV seroconversion was diagnosed in patients undergoing periodic HIV testing. Of note, three patients were diagnosed during pregnancy. The source of transmission could be identified in 52 cases. In 28 (54%) of these, a stable HIV-positive partner was identified.

At first evaluation, the Western blot test was negative in 12 patients (9%) and indeterminate in 53 (40%). In 26 of these cases, a virologic test (p24 antigen or HIV viral load) defined the diagnosis. All cases with initial negative or indeterminate Western blot had HIV infection confirmed by subsequent seroconversion. The remaining 69 (51%) patients with a reactive Western blot had a negative test within the previous six months.

The first laboratory evaluation (HIV viral load and CD4 cell count) was done at a median of 66 days (IQR 48-112) after the probable date of exposure to HIV. Median HIV-1 RNA VL was 4.87 log_10 _copies/mL (IQR 4.11-5.51) and the median absolute and percentage CD4 cell count were 479 cells/mm^3 ^(IQR 341-682) and 23% (IQR 17-28), respectively. Baseline CD4 cell counts were < 350 and < 200 cells/mm^3 ^in 27% and 6.25% of patients, respectively. A total of 42 patients (31%) started HAART during the acute phase, with a median time of 84 days (IQR 53-110), from the probable date of infection: 39 due to symptomatic infection, and in three asymptomatic cases, due to pregnancy. Since indication of HAART during PHI is considered optional in Argentina [20], the decision on whether to start treatment or not depended on the physician in charge.

### Morbidity and mortality associated with acute HIV infection

Ninety-nine patients (74%) presented with acute retroviral syndrome, lasting a median of 16 days (IQR 8-29). Twenty-six of them developed severe symptoms: seven opportunistic infections (three *Pneumocystis jiroveci *pneumonia, one histoplasmosis, one cryptococcal meningitis, one esophageal candidiasis and one pulmonary TB); nine B events (thrush, herpes zoster) and 10 non-AIDS defining severe events. The latter included aseptic meningitis, rhabdomyolysis with multi-organ failure, acute hepatitis, Bell's paralysis and guttate psoriasis.

Thirty-five patients (26.2%) required hospital admission. One patient developed chronic hydrocephaly and cognitive impairment secondary to cryptococcal meningitis and another suffered fatal disseminated histoplasmosis.

Factors associated with severe symptomatic seroconversion were CD4 cell counts lower than 350 cell/mm^3 ^(p = 0.001) and viral loads higher than 100,000 copies/mL (p = 0.001). HIV testing was requested more frequently by physicians based on clinical suspicion rather than patients' initiative (OR 5.06; 95% CI 1.83-14.04). We found no association between age, gender, birth place, risk factor or year of diagnosis with regard to severity of symptoms (Table 2).

**Table 2:** Factors associated with severe symptomatic PHI (univariate analysis) (n = 26)

Risk factor	OR (95%CI)	p
Age at seroconversion > 30 years	1.36 (0.63-2.92)	0.495

Male sex	2.52 (0.63-10.04)	0.246

Mode of HIV transmission (MSM)	1.14 (0.51-2.55)	0.58

Diagnosis based on physician suspicion	5.06 (1.83-14.04)	**< 0.001**

CD4 cell count < 350 cells/mm^3^	3.72 (1.83-7.58)	**0.001**

HIV RNA > 100,000 copies/mL	3.72 (1.58-8.77)	**0.001**

Year of diagnosis ≥ 2005	0.79 (0.37-1.70)	0.619

MSM-men who have sex with men

### 12-month morbidity and mortality

#### Untreated patients

Among the ninety-two patients who did not start HAART during acute HIV infection, 24 (26%, 95% CI: 17.5-36.3) patients presented with disease progression within the first year of infection: 12 had clinical progression (five AIDS-defining events and seven B events) and 12 exhibited immunological progression (CD4 cell count < 350 cells/mm^3^). The median time between the probable date of infection and the event presentation was 182 days (IQR 67-233). One patient who developed a non-Hodgkin lymphoma within six months of HIV infection died shortly after diagnosis.

**Figure 1:**
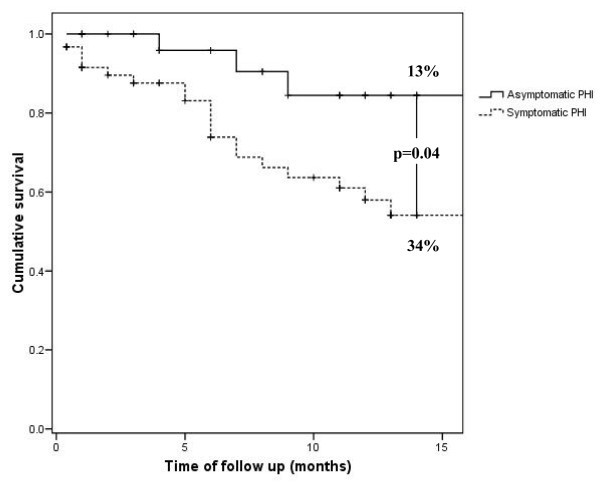
**Time to progression of HIV disease among untreated patients from the *Grupo Argentino de Seroconversión***. Progression-free survival from onset of HIV infection among untreated patients with or without symptomatic primary HIV infection.

Among untreated patients, progression was observed in 20 out of 60 symptomatic patients and in 4 out of 32 asymptomatic patients. Using Kaplan-Meier curves, estimated rates of progression at 12 months of follow up were 34% (95% CI 22.5-46.3%) among symptomatic untreated patients versus 13% (95% CI 1.1- 24.7%) in the asymptomatic group. The difference between the two curves was statistically significant (p = 0.04) (Figure 1). The hazard ratio of disease progression for untreated persons with symptomatic primary HIV infection compared with asymptomatic seroconverters was 8.44 (95% CI 0.97-73.42).

Factors associated with faster progression among untreated patients during the first year of infection were symptomatic primary HIV infection (p = 0.046), higher viral load at baseline and at six months from seroconversion (p = 0.04 and 0.008, respectively), as well as lower baseline CD4 cell count (p = 0.002). No association was found with age at seroconversion, gender, mode of HIV acquisition and year of infection. In the multivariate analysis (Table 3), only symptomatic primary HIV infection (p = 0.049) and baseline viral load higher than 5 log_10 _copies/mL (p = 0.022) remained as independent predictors of faster progression; relative risks 8.44 (95% CI 0.97-73.42) and 9.44 (95% CI 1.38-64.68), respectively. Baseline CD4 and viral load at six months were no longer associated with increased risk of progression in the multivariate model.

**Table 3:** Predictors of disease progression in untreated patients (unadjusted and adjusted analysis) (n = 92)

Risk factor	Unadjusted HR (95%CI)	p	Adjusted HR (95%CI)	p
Symptomatic PHI	1.41 (1.08- 1.83)	**0.046**	8.44 (0.97-73.42)	**0.049**

Age at seroconversion > 30 years	1.40 (0.93- 2.10)	0.159	4.42 (0.91-21.47)	0.065

Mode of HIV transmission (MSM)	1.38 (1.02-1.86)	0.081	0.99 (0.11-8.64)	0.995

Baseline CD4 cell count ≤ 350 cell/mm^3^	3.81 (1.64-8.86)	**0.002**	3.14 (0.47-20.78)	0.236

Baseline HIV RNA ≥ 100,000 copies/mL	1.91 (1.08-3.39)	**0.043**	9.44 (1.38-64.68)	**0.022**

HIV RNA at 6 months ≥ 100,000 copies/mL	9.88 (1.30-75.20)	**0.008**	2.24 (0.19-26.14)	0.520

Male sex	1.07 (0.89-1.29)	0.752	3.33 (0.16-67.54)	0.433

Year of diagnosis ≥ 2005	0.81 (0.61-1.09)	0.146	2.10 (0.20-21.99)	0.537

PHI-primary HIV infection; MSM-men who have sex with men

#### Evolution among treated patients

Among those patients who started HAART within the first 120 days of HIV infection, only three (7%) presented with HIV progression (one C event, one B event and one CD4 cell count decrease to < 350 cells/mm^3 ^despite HAART initiation) within the first year of infection. The difference to the 26% progression rate seen in the untreated group was statistically significant (p = 0.01). Of note, the C event was pulmonary TB, which is endemic in Argentina.

## Discussion

This study is the first report from the only multicentre cohort of HIV seroconverters in Argentina and one of the few descriptions of HIV-1 progression from seroconversion in Latin America.

In our cohort, the proportion of patients with symptomatic disease was similar to previous series [13,17,21,22]. Of note, one-quarter presented with serious clinical manifestations associated with seroconversion. Even though these have been previously reported [23-26], our results regarding the relatively high frequency of serious clinical manifestations during primary HIV infection are rather unusual. In our study, severe PHI was strongly associated with higher baseline viral load and low CD4 cell count, which is also consistent with other reports [27-29]. Likewise, during acute HIV infection, opportunistic infections are usually associated with low CD4 cell count. In our study, however, four out of five AIDS-defining events registered after the first 60 days of HIV infection were associated with CD4 counts greater than 200 cells/mm^3 ^(Table 4), thereby highlighting the need to consider opportunistic infection even in patients with moderate immune deficiency.

**Table 4:** AIDS-defining events during the first year of infection

Subject	Event	Time from HIV infection to event (days)	**CD4 cell count **(**cells/mm^3^)**	Outcome
1	PCP	15	27	Resolved, HAART initiated

2	PCP	15	13	Resolved, HAART initiated

3	Cryptococcal meningitis	60	227	Cognitive impairment secondary to chronic hydrocephaly

4	Disseminated histoplasmosis	32	42	Death

5	Esophageal candidiasis	9	134	Resolved, HAART initiated

6	Pulmonary TB	28	419	Resolved with TB treatment

7	PCP	25	199	Resolved, HAART initiated

8	Cytomegalovirus disease	92	278	Resolved, HAART initiated

9	Non-Hodgkin lymphoma	210	28	Death

10	Pulmonary TB	203	553	Resolved with TB treatment

11	Cryptosporidiosis	120	570	Resolved

12	Kaposi's sarcoma	230	828	Resolved, HAART and quimiotherapy initiated

PCP-*Pneumocystis jiroveci *pneumonia; TB-tuberculosis

Most of our patients were young males, with MSM being slightly overrepresented compared with the current proportion in the local HIV epidemic, where heterosexual intercourse is the most common mode of HIV transmission [9]. Greater awareness regarding acute retroviral syndrome (ARS), the higher frequency of testing among this population, and the inclusion in the cohort of a voluntary counselling and testing centre, where most of the attendants are MSM, could have influenced our results. In addition, medical prejudice could have resulted in higher recognition of ARS in MSM patients than in the heterosexual population. This could also partly explain the lower proportion of women in our cohort compared with Argentina's overall HIV population [9] (19% vs. 39%), limiting the generalization of our findings.

One-quarter of the patients who did not start HAART during the acute phase met clinical or immunological criteria (< 350 CD4 cells/mm^3^) [19,20] to initiate HAART during the first year of HIV infection. This observation is particularly relevant as one-third of the patients were already excluded in the progression analysis due to HAART initiation during the acute HIV phase, which resulted in the exclusion of a considerable proportion of symptomatic patients with risk of progression. The progression rate described here is much higher than in earlier epidemiological reports [30], which estimated a window of several years before the need for HAART initiation. However, a recent study by CASCADE cohort investigators [31] found that nearly 30% of their patients had ≤ 500 CD4 cells/mm^3 ^12 months after infection.

Symptomatic PHI and baseline HIV RNA > 100,000 copies/mL were identified in our study as predictors of disease progression in the multivariate model. These findings are consistent with prior studies [2,3,28,29,32]. While high viral loads during acute HIV infection are typically described [33,34], low plasma levels of HIV RNA have also been reported [7,35]. Comparisons across cohorts are difficult. However, an interesting finding of our study was that compared with European and North American cohorts of seroconvertors [3,4], baseline HIV RNA was higher and closer to levels seen in reports from African [8] and Asian [2] countries.

Although some differences in early laboratory values may be accounted for by differences in the quantitative methods used or the length of seroconversion intervals, first viral load measurement in our cohort was done at a median of 66 days from the probable date of infection, similar to most of the published studies [2-4,8]. There is growing evidence that initial viral load measurements, as well as the subsequent course of HIV infection, may be affected by viral [36-39] and host factors, including age, gender [40,41], race [42] and genetics [43,44].

In our cohort, the relative risk of disease progression in patients with baseline viral loads of > 100,000 copies/mL was almost 10-fold. Taking into account that more than 40% (59/134) of the patients enrolled in our cohort presented with initial viral load levels above this threshold, the impact of this finding as a prognostic factor on the subsequent course of infection deserves to be highlighted. Viral load at six months, however, did not correlate with progression; likewise, neither did CD4 cell count at baseline or six months, which underscores the need to identify other markers of progression at this early stage of infection.

Recent evidence suggesting an increase in HIV virulence over time [31,45-47] could not be corroborated, as patients who seroconverted before or after 2005 presented with similar median CD4 cell count (481 cells/mm^3 ^vs. 477 cells/mm^3^; p = NS) and disease progression (p = 0.537). However, the relatively small size of our cohort prevents us from formulating definite conclusions on this topic.

Our study has several limitations. First, it is possible that current clinical practice in Argentina limited identification to only the most symptomatic patients, which could have contributed to the faster progression seen in our cohort. In our country, universal access to HIV testing is guaranteed by law, but there are structural, social and economic barriers to access. It is estimated that at least 50% of infected people still remain unidentified [9]. Except for antenatal care, testing is usually conducted in specialized centres. HIV testing in emergency rooms, for example, is usually not accessible. These practices could have resulted in HIV testing being requested only in those patients with a more severe clinical picture, or with evident epidemiological risk. Although we cannot rule out this possibility, 26% of patients in our cohort were asymptomatic.

Second, many of the symptomatic patients started HAART during PHI, and were therefore excluded from the analysis. This could have lead to a more conservative estimate of the risk of disease progression. Third, inclusion of patients with different seroconversion intervals (i.e., acute and recent HIV infection) could have influenced our results. However, we compared rates of progression between pre- and post-seroconversion patients and found no meaningful differences (32% vs. 22%; p = 0.39).

In addition, due to the retrospective-prospective design of this study and the availability of stored blood samples only for a subset of patients enrolled after 2008, we could not study biological factors affecting immune dysregulation, such as viral tropism [39,48], specific HLA haplotypes [48,49] and regulatory T cells [50,51]. Our research group is currently conducting other studies to understand the role of these biologic factors in the course of HIV infection.

Finally, information regarding viral subtype and genotypic analysis were not available for all patients and therefore it is not presented here. It is possible that HIV subtype could influence viral load set point and subsequent course of HIV infection [36-38]. We are currently studying the potential influence of the two most prevalent subtypes of HIV-1, B and BF [52-56], on disease progression in our country.

## Conclusions

In conclusion, the data presented here have direct implications for providing HIV care in Argentina. First, acute retroviral syndrome was associated with faster progression, significant morbidity and, in some cases, with HIV-associated mortality. Therefore, awareness needs to be raised among physicians to include HIV in their differential diagnosis of febrile illness, especially in high-risk groups, such as serodiscordant couples, sexual workers, injection drug users and MSM. Likewise, HIV should be considered in any sexually active person who presents in the emergency room with flu-like syndrome as nearly 1% of them may have acute HIV infection [57,58].

Furthermore, this data should be taken into consideration when making decisions on treatment initiation. Patients with acute retroviral syndrome or high baseline viral load should be considered for treatment initiation, as our data suggest that approximately one-third of them will require treatment in the following year; new evidence also suggests benefits of earlier treatment initiation [59,60].

Combined with other ongoing research in this field, the data presented here could provide valuable information on the complex interplay between virus and host factors in HIV pathogenesis that could aid in the development of better algorithms, new therapeutic approaches and the design of preventive interventions.

## Competing interests

The authors declare that they have no competing interests.

## Authors' contributions

MES, OS, NL and PC designed the study, and analyzed and interpreted the data. MES also wrote the first draft of the manuscript. RV contributed to the design of the study. MES, OS, NL, CC, AK and PC revised the manuscript critically for important intellectual content. All authors participated in data collection, and revised and approved the final manuscript.

## References

[B1] Kaufmann GR Cunningham P Zaunders J Law M Vizzard J Carr A Cooper DA Impact of early HIV-1 RNA and T-lymphocyte dynamics during primary HIV-1 infection on the subsequent course of HIV-1 RNA levels and CD4+ T-lymphocyte counts in the first year of HIV-1 infection. Sydney Primary HIV Infection Study Group J Acquir Immune Defic Syndr 1999 14 437 444 10961604 10.1097/00126334-199912150-00003

[B2] Buchacz K Hu DJ Vanichseni S Mock PA Chaowanachan T Srisuwanvilai LO Gvetadze R Van Griensven F Tappero JW Kitayaporn D Kaewkungwal J Choopanya K Mastro TD Early markers of HIV-1 disease progression in a prospective cohort of seroconverters in Bangkok, Thailand: implications for vaccine trials J Acquir Immune Defic Syndr 2004 14 853 860 10.1097/00126334-200407010-00013 15213570

[B3] Hubert JB Burgard M Dussaix E Tamalet C Deveau C Le Chenadec J Chaix ML Marchadier E Vilde JL Delfraissy JF Meyer L Rouzioux C Natural history of serum HIV-1 RNA levels in 330 patients with a known date of infection. The SEROCO Study Group Aids 2000 14 123 131 10.1097/00002030-200001280-00007 10708282

[B4] Lyles RH Munoz A Yamashita TE Bazmi H Detels R Rinaldo CR Margolick JB Phair JP Mellors JW Natural history of human immunodeficiency virus type 1 viremia after seroconversion and proximal to AIDS in a large cohort of homosexual men. Multicenter AIDS Cohort Study J Infect Dis 2000 14 872 880 10.1086/315339 10720507

[B5] Schiffer V Deveau C Meyer L Iraqui I Nguyen-Wartel A Chaix ML Delfraissy JF Rouzioux C Venet A Goujard C Recent changes in the management of primary HIV-1 infection: results from the French PRIMO cohort HIV Med 2004 14 326 333 10.1111/j.1468-1293.2004.00231.x 15369507

[B6] Rangsin R Chiu J Khamboonruang C Sirisopana N Eiumtrakul S Brown AE Robb M Beyrer C Ruangyuttikarn C Markowitz LE Nelson KE The natural history of HIV-1 infection in young Thai men after seroconversion J Acquir Immune Defic Syndr 2004 14 622 629 10.1097/00126334-200405010-00011 15097306

[B7] Djomand G Duerr A Faulhaber JC Struchiner CJ Pacheco AG Barroso PF Melo MF Schechter M Viral load and CD4 count dynamics after HIV-1 seroconversion in homosexual and bisexual men in Rio de Janeiro, Brazil J Acquir Immune Defic Syndr 2006 14 401 404 10.1097/01.qai.0000243117.21788.90 17031316

[B8] Salamon R Marimoutou C Ekra D Minga A Nerrienet E Huet C Gourvellec G Bonard D Coulibaly I Combe P Dabis F Bondurand A Montagnier L Clinical and biological evolution of HIV-1 seroconverters in Abidjan, Cote d'Ivoire, 1997-2000 J Acquir Immune Defic Syndr 2002 14 149 157 11832684 10.1097/00042560-200202010-00007

[B9] Argentinean National Health Ministry AIDS and STD Department26th Bulletin of HIV-AIDS in Argentina 2009 Buenos Aires

[B10] Socías ME Describing the acute HIV infection in Argentina: preliminary results of the Grupo Argentino de Seroconversion. 4th International Workshop on HIV Transmission Reviews in Antiviral Therapy 2009 14 S12

[B11] Sued O Laufer N Amante L Remondegui C Lazaro M Zala C Cangelosi D Castelli J Cabrini M Figueroa MI Duarte A Socías ME Rolon M Crudo F Garda E Gun A Krolewiecki A Gomez-Carrillo M Salomón H Zapatella M Perez H Cahn P Baseline characteristics in HIV primary infections in Argentina: multicentric study [THPE0085] XVII International AIDS Conference 3-8 August 2008; Mexico City

[B12] Interpretation and use of the western blot assay for serodiagnosis of human immunodeficiency virus type 1 infections MMWR Morb Mortal Wkly Rep 1989 14 1 7 2501638

[B13] Cooper DA Gold J Maclean P Donovan B Finlayson R Barnes TG Michelmore HM Brooke P Penny R Acute AIDS retrovirus infection. Definition of a clinical illness associated with seroconversion Lancet 1985 14 537 540 2857899 10.1016/s0140-6736(85)91205-x

[B14] Kassutto S Rosenberg ES Primary HIV type 1 infection Clin Infect Dis 2004 14 1447 1453 10.1086/420745 15156484

[B15] 1993 revised classification system for HIV infection and expanded surveillance case definition for AIDS among adolescents and adults MMWR Recomm Rep 1992 14 1 19 1361652

[B16] Lewden C Thiebaut R Boufassa F Coulibaly A Malateste K Seng R Toni TD Inwoley A Rouzioux C Minga A Anglaret X Meyer L Comparison of Early CD4 T-Cell Count in HIV-1 Seroconverters in Cote d'Ivoire and France: The ANRS PRIMO-CI and SEROCO Cohorts J Acquir Immune Defic Syndr 2009 10.1097/QAI.0b013e3181b84260 19745754

[B17] Sued O Miro JM Alquezar A Claramonte X Garcia F Plana M Arnedo M de Lazzari E Gil C Manzardo C Blanco JL Martinez E Mallolas J Joseph J Pumarola T Gallart T Gatell JM Primary human immunodeficiency virus type 1 infection: clinical, virological and immunological characteristics of 75 patients (1997-2003) Enferm Infecc Microbiol Clin 2006 14 238 244 10.1016/S0213-005X(06)73769-7 16725083

[B18] Goujard C Bonarek M Meyer L Bonnet F Chaix ML Deveau C Sinet M Galimand J Delfraissy JF Venet A Rouzioux C Morlat P CD4 cell count and HIV DNA level are independent predictors of disease progression after primary HIV type 1 infection in untreated patients Clin Infect Dis 2006 14 709 715 10.1086/500213 16447119

[B19] World Health Organization Antiretroviral therapy for HIV infection in adults and adolescents: recommendations for a public health approach 2010 revision 2010 WHO 23741771

[B20] Sociedad Argentina de Infectología III Argentinean Consensus of Antirretroviral Therapy 2010 Buenos Aires

[B21] Schacker T Collier AC Hughes J Shea T Corey L Clinical and epidemiologic features of primary HIV infection Ann Intern Med 1996 14 257 264 8678387 10.7326/0003-4819-125-4-199608150-00001

[B22] Hightow-Weidman LB Golin CE Green K Shaw EN Macdonald PD Leone PA Identifying People with Acute HIV Infection: Demographic Features, Risk Factors, and Use of Health Care among Individuals with AHI in North Carolina AIDS Behav 2009 10.1007/s10461-008-9519-5 PMC2787774 19127422

[B23] Lillie PJ Barlow GD Moss PJ Parsonage MJ Adams K Thaker HK HIV seroconversion complicated by Mycobacterium kansasii infection Aids 2007 14 650 652 10.1097/QAD.0b013e32803277e9 17314530

[B24] Signorini L Gulletta M Coppini D Donzelli C Stellini R Manca N Carosi G Matteelli A Fatal disseminated toxoplasmosis during primary HIV infection Curr HIV Res 2007 14 273 274 10.2174/157016207780077011 17346141

[B25] Szabo S James CW Telford G Unusual presentations of primary human immunodeficiency virus infection AIDS Patient Care STDS 2002 14 251 254 10.1089/10872910260066688 12133260

[B26] Tattevin P Camus C Arvieux C Ruffault A Michelet C Multiple organ failure during primary HIV infection Clin Infect Dis 2007 14 e28 29 10.1086/510683 17205433

[B27] Lavreys L Baeten JM Overbaugh J Panteleeff DD Chohan BH Richardson BA Mandaliya K Ndinya-Achola JO Kreiss JK Virus load during primary Human Immunodeficiency Virus (HIV) type 1 infection is related to the severity of acute HIV illness in Kenyan women Clin Infect Dis 2002 14 77 81 10.1086/340862 12060878

[B28] Kelley CF Barbour JD Hecht FM The relation between symptoms, viral load, and viral load set point in primary HIV infection J Acquir Immune Defic Syndr 2007 14 445 448 10.1097/QAI.0b013e318074ef6e 17514014

[B29] Vanhems P Lambert J Cooper DA Perrin L Carr A Hirschel B Vizzard J Kinloch-de Loes S Allard R Severity and prognosis of acute human immunodeficiency virus type 1 illness: a dose-response relationship Clin Infect Dis 1998 14 323 329 10.1086/516289 9502449

[B30] Munoz A Wang MC Bass S Taylor JM Kingsley LA Chmiel JS Polk BF Acquired immunodeficiency syndrome (AIDS)-free time after human immunodeficiency virus type 1 (HIV-1) seroconversion in homosexual men. Multicenter AIDS Cohort Study Group Am J Epidemiol 1989 14 530 539 2669471 10.1093/oxfordjournals.aje.a115367

[B31] Lodi S Porter K Philips A Time to reaching CD4 ≤ 500 for individuals followed-up since HIV seroconversion [MOPEB050] 5th IAS Conference on HIV Pathogenesis, Treatment and Prevention; 19-22 July 2009 Cape Town, South Africa

[B32] Lavreys L Baeten JM Chohan V McClelland RS Hassan WM Richardson BA Mandaliya K Ndinya-Achola JO Overbaugh J Higher set point plasma viral load and more-severe acute HIV type 1 (HIV-1) illness predict mortality among high-risk HIV-1-infected African women Clin Infect Dis 2006 14 1333 1339 10.1086/503258 16586394

[B33] Rosenberg ES Altfeld M Poon SH Phillips MN Wilkes BM Eldridge RL Robbins GK D'Aquila RT Goulder PJ Walker BD Immune control of HIV-1 after early treatment of acute infection Nature 2000 14 523 526 10.1038/35035103 11029005

[B34] Little SJ McLean AR Spina CA Richman DD Havlir DV Viral dynamics of acute HIV-1 infection J Exp Med 1999 14 841 850 10.1084/jem.190.6.841 10499922 PMC2195636

[B35] Rinke de Wit TF Tsegaye A Wolday D Hailu B Aklilu M Sanders E Hagos M Kliphuis A Pollakis G Krol A Geskus R Miedema F Goudsmit J Coutinho R Fontanet AL Primary HIV-1 subtype C infection in Ethiopia J Acquir Immune Defic Syndr 2002 14 463 470 10.1097/00126334-200208150-00001 12154336

[B36] Santoro-Lopes G Harrison LH Tavares MD Xexeo A Dos Santos AC Schechter M HIV disease progression and V3 serotypes in Brazil: is B different from B-Br? AIDS Res Hum Retroviruses 2000 14 953 958 10.1089/08892220050058362 10890356

[B37] Kiwanuka N Laeyendecker O Robb M Kigozi G Arroyo M McCutchan F Eller LA Eller M Makumbi F Birx D Wabwire-Mangen F Serwadda D Sewankambo NK Quinn TC Wawer M Gray R Effect of human immunodeficiency virus Type 1 (HIV-1) subtype on disease progression in persons from Rakai, Uganda, with incident HIV-1 infection J Infect Dis 2008 14 707 713 10.1086/527416 18266607

[B38] Kanki PJ Hamel DJ Sankale JL Hsieh C Thior I Barin F Woodcock SA Gueye-Ndiaye A Zhang E Montano M Siby T Marlink R I ND Essex ME S MB Human immunodeficiency virus type 1 subtypes differ in disease progression J Infect Dis 1999 14 68 73 10.1086/314557 9841824

[B39] Daar ES Kesler KL Petropoulos CJ Huang W Bates M Lail AE Coakley EP Gomperts ED Donfield SM Baseline HIV type 1 coreceptor tropism predicts disease progression Clin Infect Dis 2007 14 643 649 10.1086/520650 17683002

[B40] Sterling TR Vlahov D Astemborski J Hoover DR Margolick JB Quinn TC Initial plasma HIV-1 RNA levels and progression to AIDS in women and men N Engl J Med 2001 14 720 725 10.1056/NEJM200103083441003 11236775

[B41] Donnelly CA Bartley LM Ghani AC Le Fevre AM Kwong GP Cowling BJ van Sighem AI de Wolf F Rode RA Anderson RM Gender difference in HIV-1 RNA viral loads HIV Med 2005 14 170 178 10.1111/j.1468-1293.2005.00285.x 15876283

[B42] Anastos K Gange SJ Lau B Weiser B Detels R Giorgi JV Margolick JB Cohen M Phair J Melnick S Rinaldo CR Kovacs A Levine A Landesman S Young M Munoz A Greenblatt RM Association of race and gender with HIV-1 RNA levels and immunologic progression J Acquir Immune Defic Syndr 2000 14 218 226 10969345 10.1097/00126334-200007010-00004

[B43] Saah AJ Hoover DR Weng S Carrington M Mellors J Rinaldo CR Jr Mann D Apple R Phair JP Detels R O'Brien S Enger C Johnson P Kaslow RA Association of HLA profiles with early plasma viral load, CD4+ cell count and rate of progression to AIDS following acute HIV-1 infection. Multicenter AIDS Cohort Study Aids 1998 14 2107 2113 10.1097/00002030-199816000-00005 9833851

[B44] Altfeld M Addo MM Rosenberg ES Hecht FM Lee PK Vogel M Yu XG Draenert R Johnston MN Strick D Allen TM Feeney ME Kahn JO Sekaly RP Levy JA Rockstroh JK Goulder PJ Walker BD Influence of HLA-B57 on clinical presentation and viral control during acute HIV-1 infection Aids 2003 14 2581 2591 10.1097/00002030-200312050-00005 14685052

[B45] Crum-Cianflone N Eberly L Zhang Y Ganesan A Weintrob A Marconi V Barthel RV Fraser S Agan BK Wegner S Is HIV becoming more virulent? Initial CD4 cell counts among HIV seroconverters during the course of the HIV epidemic: 1985-2007 Clin Infect Dis 2009 14 1285 1292 10.1086/597777 19309306 PMC2746018

[B46] Dorrucci M Rezza G Porter K Phillips A Temporal trends in postseroconversion CD4 cell count and HIV load: the Concerted Action on Seroconversion to AIDS and Death in Europe Collaboration, 1985-2002 J Infect Dis 2007 14 525 534 10.1086/510911 17230412

[B47] Muller V Maggiolo F Suter F Ladisa N De Luca A Antinori A Sighinolfi L Quiros-Roldan E Carosi G Torti C Increasing clinical virulence in two decades of the Italian HIV epidemic PLoS Pathog 2009 14 e1000454 10.1371/journal.ppat.1000454 19478880 PMC2682199

[B48] Dalmau J Puertas MC Azuara M Marino A Frahm N Mothe B Izquierdo-Useros N Buzon MJ Paredes R Matas L Allen TM Brander C Rodrigo C Clotet B Martinez-Picado J Contribution of immunological and virological factors to extremely severe primary HIV type 1 infection Clin Infect Dis 2009 14 229 238 10.1086/595704 19093810 PMC2806184

[B49] O'Brien SJ Gao X Carrington M HLA and AIDS: a cautionary tale Trends Mol Med 2001 14 379 381 10.1016/S1471-4914(01)02131-1 11530315

[B50] Cao W Jamieson BD Hultin LE Hultin PM Detels R Regulatory T cell expansion and immune activation during untreated HIV type 1 infection are associated with disease progression AIDS Res Hum Retroviruses 2009 14 183 191 10.1089/aid.2008.0140 19239357 PMC2782619

[B51] Kared H Lelievre JD Donkova-Petrini V Aouba A Melica G Balbo M Weiss L Levy Y HIV-specific regulatory T cells are associated with higher CD4 cell counts in primary infection Aids 2008 14 2451 2460 10.1097/QAD.0b013e328319edc0 19005268 PMC3195674

[B52] Dilernia DA Gomez AM Lourtau L Marone R Losso MH Salomon H Gomez-Carrillo M HIV type 1 genetic diversity surveillance among newly diagnosed individuals from 2003 to 2005 in Buenos Aires, Argentina AIDS Res Hum Retroviruses 2007 14 1201 1207 10.1089/aid.2007.0068 17961105

[B53] Masciotra S Livellara B Belloso W Clara L Tanuri A Ramos AC Baggs J Lal R Pieniazek D Evidence of a high frequency of HIV-1 subtype F infections in a heterosexual population in Buenos Aires, Argentina AIDS Res Hum Retroviruses 2000 14 1007 1014 10.1089/08892220050058425 10890362

[B54] Thomson MM Villahermosa ML Vazquez-de-Parga E Cuevas MT Delgado E Manjon N Medrano L Perez-Alvarez L Contreras G Carrillo MG Salomon H Najera R Widespread circulation of a B/F intersubtype recombinant form among HIV-1-infected individuals in Buenos Aires, Argentina Aids 2000 14 897 899 10.1097/00002030-200005050-00020 10839601

[B55] Avila MM Pando MA Carrion G Peralta LM Salomon H Carrillo MG Sanchez J Maulen S Hierholzer J Marinello M Negrete M Russell KL Carr JK Two HIV-1 epidemics in Argentina: different genetic subtypes associated with different risk groups J Acquir Immune Defic Syndr 2002 14 422 426 11917249 10.1097/00126334-200204010-00015

[B56] Petroni A Deluchi G Pryluka D Rotryng F Bortolozzi R Lopardo G Bouzas MB Zapiola I Garone D Rodriguez C Chiocconi E Lazaro ME Murano F Maranzana A Oliva SM Aparicio M Beltran M Benetucci JA Update on primary HIV-1 resistance in Argentina: emergence of mutations conferring high-level resistance to nonnucleoside reverse transcriptase inhibitors in drug-naive patients J Acquir Immune Defic Syndr 2006 14 506 510 10.1097/01.qai.0000222285.44460.e2 16773027

[B57] Pincus JM Crosby SS Losina E King ER LaBelle C Freedberg KA Acute human immunodeficiency virus infection in patients presenting to an urban urgent care center Clin Infect Dis 2003 14 1699 1704 10.1086/379772 14689354

[B58] Rosenberg ES Caliendo AM Walker BD Acute HIV infection among patients tested for mononucleosis N Engl J Med 1999 14 969 10094651 10.1056/nejm199903253401217

[B59] Kitahata MM Gange SJ Abraham AG Merriman B Saag MS Justice AC Hogg RS Deeks SG Eron JJ Brooks JT Rourke SB Gill MJ Bosch RJ Martin JN Klein MB Jacobson LP Rodriguez B Sterling TR Kirk GD Napravnik S Rachlis AR Calzavara LM Horberg MA Silverberg MJ Gebo KA Goedert JJ Benson CA Collier AC Van Rompaey SE Crane HM McKaig RG Lau B Freeman AM Moore RD Effect of early versus deferred antiretroviral therapy for HIV on survival N Engl J Med 2009 14 1815 1826 10.1056/NEJMoa0807252 19339714 PMC2854555

[B60] Sterne JA May M Costagliola D de Wolf F Phillips AN Harris R Funk MJ Geskus RB Gill J Dabis F Miro JM Justice AC Ledergerber B Fatkenheuer G Hogg RS Monforte AD Saag M Smith C Staszewski S Egger M Cole SR Timing of initiation of antiretroviral therapy in AIDS-free HIV-1-infected patients: a collaborative analysis of 18 HIV cohort studies Lancet 2009 14 1352 1363 19361855 10.1016/S0140-6736(09)60612-7 PMC2670965

